# Network Analysis of a *Pkd1*-Mouse Model of Autosomal Dominant Polycystic Kidney Disease Identifies HNF4α as a Disease Modifier

**DOI:** 10.1371/journal.pgen.1003053

**Published:** 2012-11-29

**Authors:** Luis F. Menezes, Fang Zhou, Andrew D. Patterson, Klaus B. Piontek, Kristopher W. Krausz, Frank J. Gonzalez, Gregory G. Germino

**Affiliations:** 1 National Institute of Diabetes and Digestive and Kidney Diseases, National Institutes of Health, Bethesda, Maryland, United States of America; 2 Laboratory of Metabolism, Center for Cancer Research, National Cancer Institute, National Institutes of Health, Bethesda, Maryland, United States of America; 3 The Johns Hopkins University School of Medicine, Baltimore, Maryland, United States of America; Harvard Medical School, United States of America

## Abstract

Autosomal Dominant Polycystic Kidney Disease (ADPKD; MIM ID's 173900, 601313, 613095) leads to end-stage kidney disease, caused by mutations in *PKD1* or *PKD2*. Inactivation of *Pkd1* before or after P13 in mice results in distinct early- or late-onset disease. Using a mouse model of ADPKD carrying floxed *Pkd1* alleles and an inducible Cre recombinase, we intensively analyzed the relationship between renal maturation and cyst formation by applying transcriptomics and metabolomics to follow disease progression in a large number of animals induced before P10. Weighted gene co-expression network analysis suggests that *Pkd1*-cystogenesis does not cause developmental arrest and occurs in the context of gene networks similar to those that regulate/maintain normal kidney morphology/function. Knowledge-based Ingenuity Pathway Analysis (IPA) software identifies HNF4α as a likely network node. These results are further supported by a meta-analysis of 1,114 published gene expression arrays in *Pkd1* wild-type tissues. These analyses also predict that metabolic pathways are key elements in postnatal kidney maturation and early steps of cyst formation. Consistent with these findings, urinary metabolomic studies show that *Pkd1* cystic mutants have a distinct profile of excreted metabolites, with pathway analysis suggesting altered activity in several metabolic pathways. To evaluate their role in disease, metabolic networks were perturbed by inactivating *Hnf4α* and *Pkd1*. The *Pkd1/Hnf4α* double mutants have significantly more cystic kidneys, thus indicating that metabolic pathways could play a role in *Pkd1*-cystogenesis.

## Author Summary

Autosomal Dominant Polycystic Kidney Disease (ADPKD) is the most common genetic cause of polycystic kidney disease and is responsible for 4.6% of the end-stage renal disease (ESRD) cases in the United States. It is most often caused by mutation in the *PKD1* gene. To understand this disease, we made a mouse model in which we could delete the *Pkd1* gene and study the animal as its kidney becomes cystic. Using this model, we had previously found that the maturation status of the animal determines whether cysts form within days or within months, and we had narrowed down this switch to a two-day interval. In the current study, we used the rapid cyst-forming model to analyze the expression pattern of thousands of genes in mutant and control kidneys, and metabolites excreted in the urine. Our results identify a number of genes that may be involved in cyst formation and suggest that metabolic changes may play a role in ADPKD and could alter disease progression. These analyses also predict that metabolic pathways are key elements in normal postnatal kidney maturation.

## Introduction

Autosomal Dominant Polycystic Kidney Disease (ADPKD; MIM ID's 173900, 601313, 613095) is the most common genetic cause of polycystic kidney disease (PKD), with an estimated prevalence of approximately 1∶1000 [1]. ADPKD is caused by mutations in *PKD1* (∼85%) or *PKD2* (∼15%), which encode polycystin-1 (PC1) and polycystin-2 (PC2), respectively. Morphologically, the disease is characterized by the gradual replacement of normal kidney parenchyma by fluid-filled cysts [2]. Though 15 years have elapsed since the identification of *PKD1* and *PKD2* and despite intense effort focused on determining the function of their respective gene products, the pathways and mechanisms by which PC1 and PC2 regulate luminal diameter remain poorly understood.

To better model the disease in rodents and determine how acquired *Pkd1* inactivation results in cyst formation, we had developed a novel mouse line with floxed alleles of *Pkd1* that could be conditionally inactivated in a large proportion of kidney cells at distinct timepoints [3], [4]. We used this line to determine that *Pkd1* inactivation prior to P12 results in cyst formation within 7–21 days, whereas inactivation on or after P14 results in cyst formation only after 4–5 months [4]. This effect is associated with changes in gene expression patterns that take place between P12 and P14 in wild-type kidneys. Other groups have similarly identified developmental windows in which inactivation of *Pkd1* [5] or other cystogenes (Kif3a and Tg737 [6], HNF-1β [7]) leads to early- or late-onset cyst development. These observations suggest that pathways related to kidney maturation play relevant roles in rapid cyst formation.

The existence of a functional maturation phase in postnatal kidney was postulated over 50 years ago, when physiologists observed that the composition and volume of urine excreted by newborn animals, when compared to adults, tended to vary less in response to osmotic stress [8]. Subsequent studies linked some of these changes to intrinsic renal postnatal events, such as changes in expression patterns of tight junction proteins or transporters [9], [10]. However, the pathways responsible for postnatal kidney maturation and how they relate to the maintenance and establishment of kidney architecture remain unknown. The current study tackles this issue by analyzing mRNA, microRNA and urinary metabolomics during the early stages of cyst formation and kidney maturation in control and mutant animals induced before P10. To our knowledge, this is the first study to focus on this short time interval and characterize the interplay between these two phenomena. Our results suggest that there is no developmental arrest in PKD kidneys, but that metabolic and HNF4α-related pathways involved in kidney maturation are modifiers of cystogenesis. This finding was confirmed by showing that double knockout of *Hnf4α* and *Pkd1* significantly worsens cystic disease.

## Results

### Metabolic Pathways Could Underlie Kidney Maturation and Determine the Susceptibility to Rapid Cyst Formation in the Early-Onset Model of ADPKD

**Figure 1:**
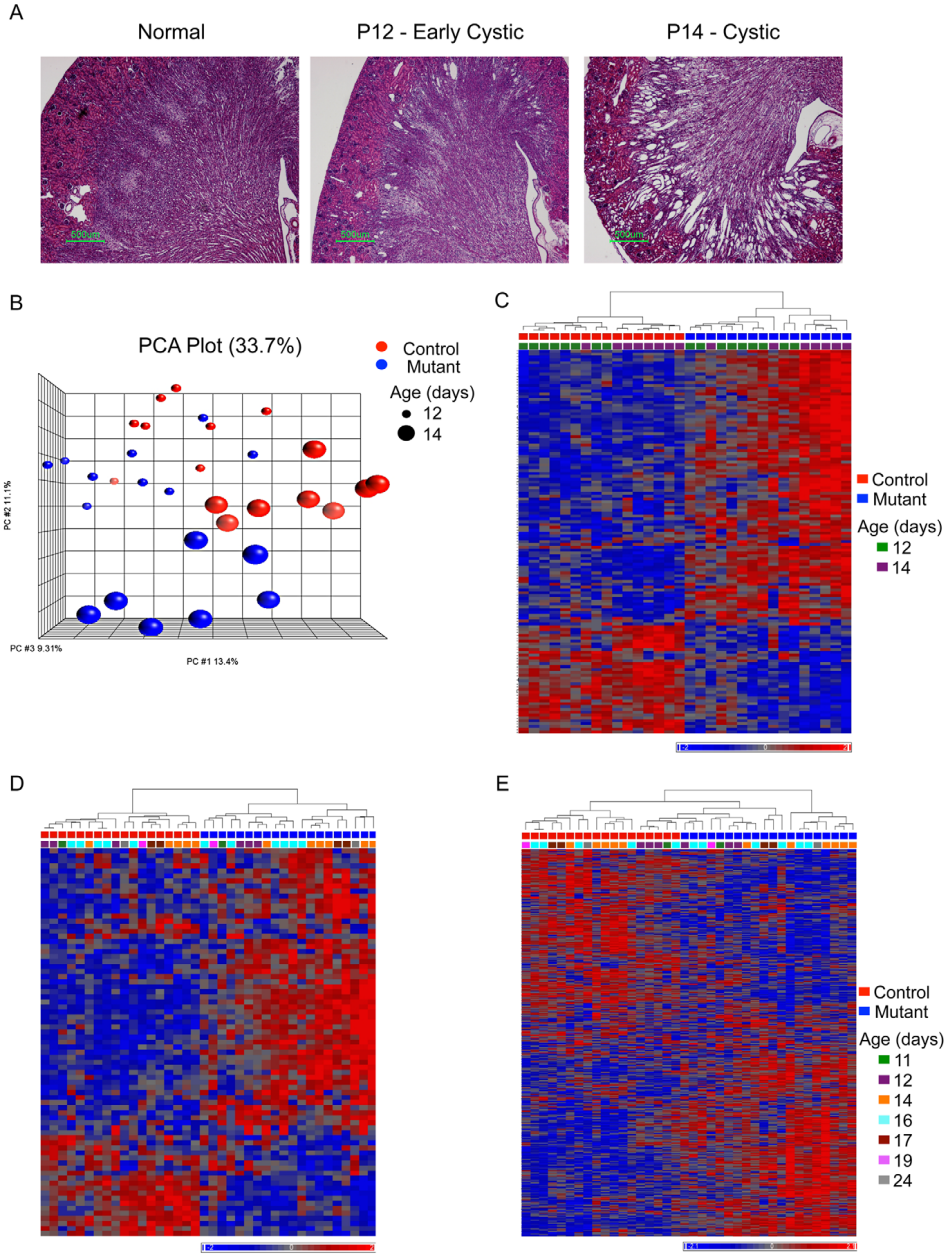
Histology and gene expression patterns in early-onset model. A) Representative kidney histology (H&E stain; scale bar: 500 mm); B) PCA plot: genotype and age explain most of the clustering in the complete set of 70 kidneys in the early-onset model (control: red; mutant: blue; size of spheres: age). C and D) Heatmap plot of mutant-signature (genes in Table S1) in the test (C) and validation (D) groups. E) Heatmap plot of ME2 (genes in Table S3) cluster in the validation group.

A total of 70 kidneys [36 *Pkd1^cko/cko^*;tamoxifen-cre positive (mutant) and 34 *Pkd1^cko/cko^*;tamoxifen-cre negative (control)] in which *Pkd1* knockout was induced between P5 and P9 were harvested between P11 and P24 and analyzed using gene expression arrays. This set includes mutant kidneys with variable degrees of cystic transformation. In fact, animals induced at P7 display mostly normal histology at P12 (6 normal and 11 with dilated tubes) and are cystic at P14 (4 dilated and 14 grossly cystic; Figure 1A). Consistent with our previous data showing high rates of *Pkd1* inactivation in this model [4], mutant kidneys are enlarged and globally cystic at later time points.

A subset of 32 P12 and P14 mutant and control samples was used for bioinformatics analysis (test group), and the results were validated using the remaining samples (validation group). Principal component analysis (PCA) plots show that clustering correlates with genotype and age in the test group (Figure 1B). Statistical analysis shows that few genes (48 probes; fold change>1.2 and fdr-adjusted p<0.05) are different between mutants and controls at P12, but that over 600 genes (622 probes; fold change>1.2 and fdr-adjusted p<0.05) are differentially expressed at P14. To enrich for genes differentially expressed at the early stages of cyst formation, we focused on the subset that had some evidence of differential expression at P12 (i.e. either fold change>1.2 or fdr-adjusted p<0.05) and that fulfilled both criteria at P14 (i.e. fold change>1.2 and fdr-adjusted p<0.05). This yields a mutant-signature of 87 genes (Table S1). Most of these genes are up-regulated in mutants, with higher expression levels in cystic (P14) animals (Figure 1C). The mutant-signature also clusters mutant and control mice in the validation group (Figure 1D). Gene ontology (GO) classification suggests that the mutant-signature is significantly enriched in categories related to cell differentiation (3.64E-05), tissue remodeling (5.75E-05), and a variety of developmental and metabolic processes (E-04 and E-03) (Table S2).

One surprising result was the small number of genes with changes greater than 50%. Despite this finding, the expression patterns in mutant and control groups are sufficiently different to cluster most samples according to genotype, suggesting many small, but genotype-specific, differences. Weighted gene co-expression network analysis (WGCNA) is an approach particularly well suited for such situations. WGCNA can be used to identify clusters of genes with similar patterns of expression across a set of microarray samples rather than individual, differentially expressed genes [11]. The approach provides various features that can be used to cluster closely related patterns into a small number of distinct modules, each of which can be summarized by its first principal component (module eigengene), which can then be associated with genotype, developmental state or phenotypic traits. The greatly reduced number of variables also simplifies the analyses by minimizing the multiple testing problem. Using WGCNA, one can create a connectivity matrix that models gene networks with hubs, a condition likely met by biological networks [12].

**Figure 2:**
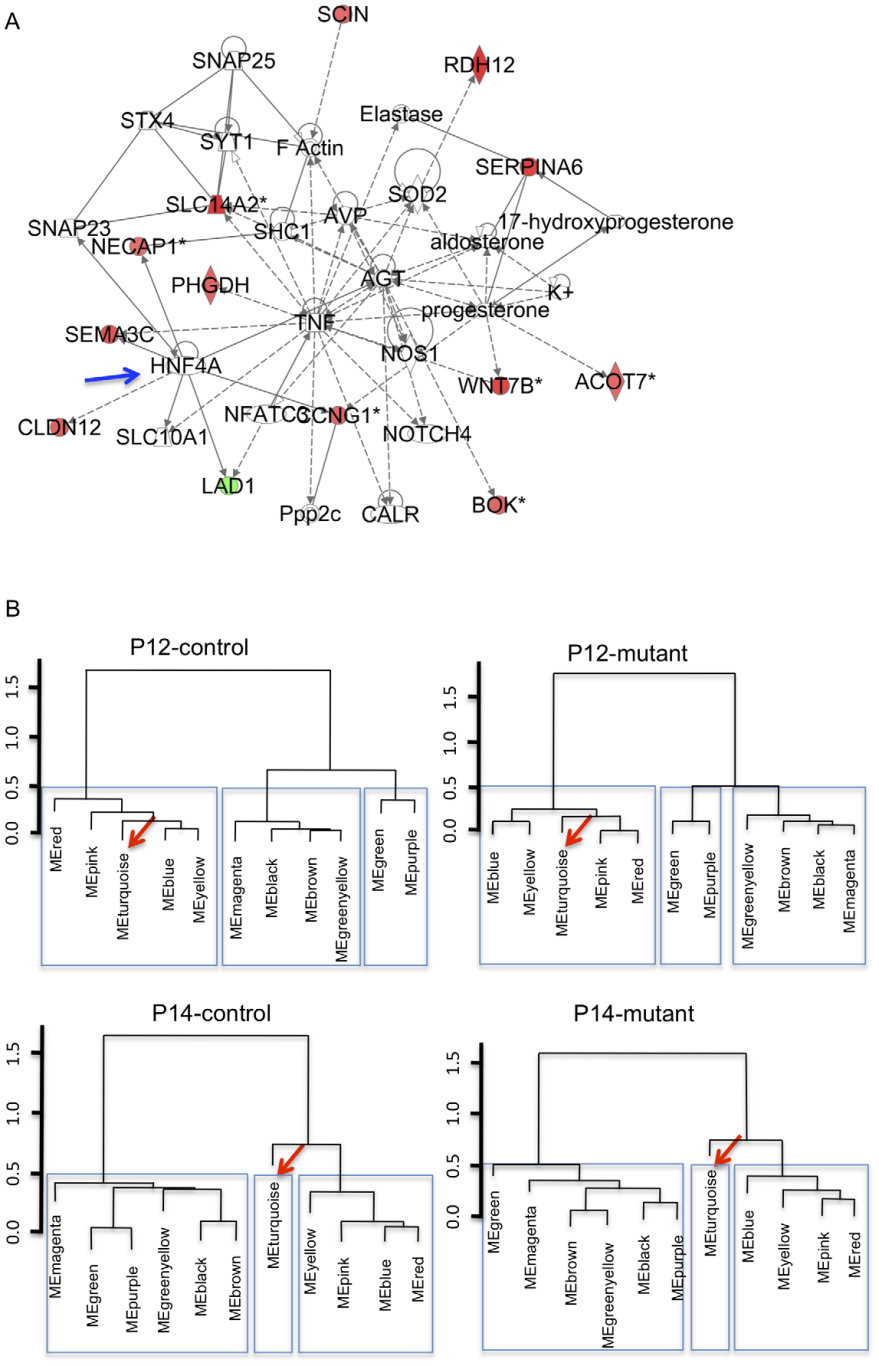
Metabolic pathways could underlie kidney maturation and determine the susceptibility to rapid cyst formation in the early-onset model of ADPKD. A) Knowledge-based IPA network (score: 24) using genes in early-onset mutant-signature (genes in Table S1). HNF4α(blue arrow) is a node (green/red: genes down/up-regulated in P12 mutant kidneys; solid lines: direct interactions; dashed lines: indirect interactions). B) Dendrograms of module eigengenes showing blocks of correlated eigengenes (meta-modules: in rectangles) suggest that gene correlation networks are preserved in mutant animals but change during P12 to P14 kidney maturation. The metabolism-related MEturquoise cluster (arrow; genes in Table S5) is part of meta-module at P12 but not at P14.

We therefore used WGCNA to analyze our dataset. Calculating the correlation between module eigengenes and genotypes, we identified one significantly correlated cluster (ME2, 629 genes, correlation: 0.81, p = 2.5E-08; Table S3). Of the 629 genes in ME2, 67 are part of the mutant-signature described above (overlap p = 6.0E-20). Furthermore, this cluster distinguishes mutant and control groups also in the validation set of samples (Figure 1E). The ME2 cluster also was enriched for multiple GO categories related to development (E-08), cell differentiation, anatomical structure morphogenesis (E-07), and metabolic pathways E-04; Table S4). Additional pathway analysis using the knowledge-based Ingenuity Pathway Analysis (IPA) software suggested that several genes in both ME2 and the mutant signature gene sets not only belong to metabolic functional groups but are also connected to/via HNF4α, a transcription factor involved in the regulation of several metabolic and developmental pathways [13] (Figure 2A). Interestingly, other nodes previously implicated in PKD, such as Tumor Necrosis Factor (TNF) [14], angiotensinogen (AGT) [15] and arginine vasopressin (AVP) [16] were also identified in this network.

### Gene Correlation Networks Are Preserved in Mutant Animals

To further analyze the link between kidney maturation and early stages of cyst formation, consensus gene modules (i.e. clusters of genes that are highly connected in each of the four experimental groups: (P12/P14) x (mutant/control)) were identified using WGCNA. Correlations between module eigengenes were then calculated for each experimental group [17]. The analysis of the preservation of the correlation between module eigengenes across different biological conditions has been proposed as a measure of similarity between gene networks [18], and in theory allows one to determine if changes in gene expression patterns are due to changes in gene networks, or to changes in the levels of expression within conserved gene networks.

Our data yield a high degree of preservation between the various gene modules across all conditions (preservation values: 0.88 to 0.92), suggesting no major network changes. In addition, using a dendrogram to depict eigengene correlations, sets of highly correlated eigengenes (i.e. meta-modules) can be readily visualized (Figure 2B) and are mostly preserved between mutant and controls. However, the turquoise module (Table S5), which is highly correlated with the pink/blue/yellow/red modules at P12, is less correlated at P14, both in mutants and controls (Figure 2B). These data suggest the surprising result that the major change in how the gene networks are inter-related in our model is not due to *Pkd1* inactivation, but rather due to changes that occur normally between P12 and P14. They also imply that the transcriptional networks activated during normal kidney maturation are also deployed in the mutant kidney, which, our data suggest, mature normally. These data are also consistent with the PCA plot in Figure 1B, which separated P12 and P14 animals.

Given the striking difference in the susceptibility to the effects of *Pkd1* inactivation before or after P13, it is therefore likely that the biological processes modulated by genes in the turquoise module are important modifiers of PKD. Gene ontology classification suggests that MEturquoise is almost exclusively enriched for metabolic pathways (top hit, generation of energy, p-2.57E-21; Table S6). Taken together with the enrichment of metabolic pathways in disease-specific gene sets (ME2 and mutant signature), our data are consistent with a role of the metabolic contexts in determining the switch from early- to late-onset cystic disease in this *Pkd1* model. These findings suggest the unintuitive result that metabolic, rather than developmental, pathways are responsible for the dramatic change in susceptibility to *Pkd1* inactivation in P12 vs. P14 mice.

### No Evidence of MicroRNA Changes in *Pkd1* Mutant Kidneys

**Figure 3:**
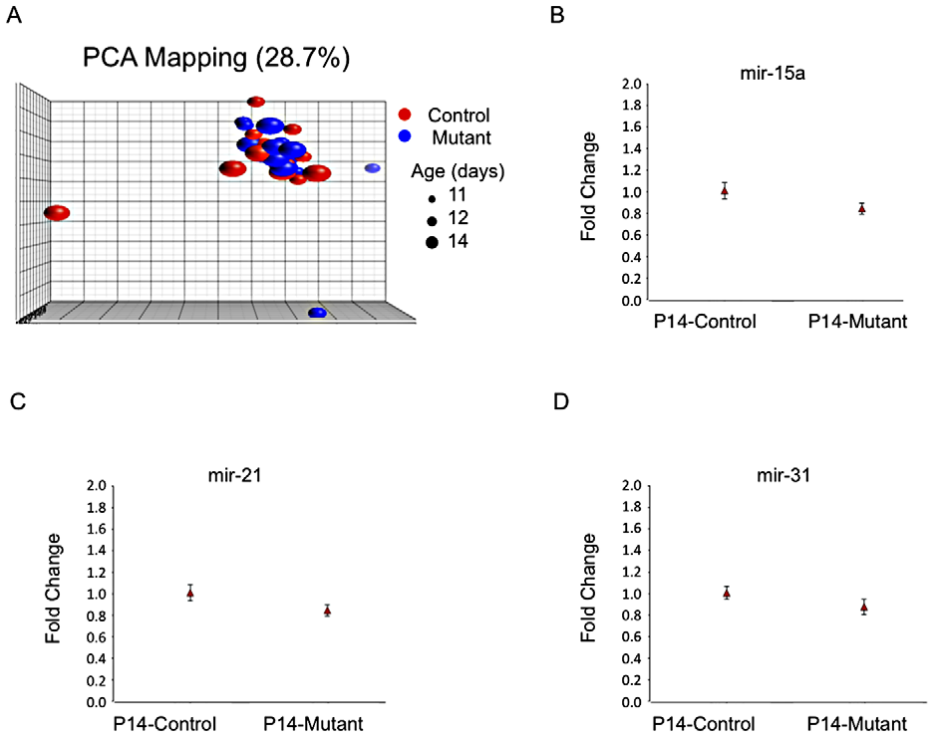
No evidence for distinct microRNA expression patterns. A) PCA plot of microRNA expression covariance matrix showing absence of clustering according to mutant- or age-specific patterns (microRNAs in Table S7). B to D) Plots of fold-change (to control) of RT-PCR data showing similar expression levels of mir-15a (B), mir-21 (C) and mir-31 (D; P14 control kidneys: 5 samples, 4 replicates each; P14 mutant kidneys: 6 samples, 4 replicates each).

MicroRNA's are thought to play an important role in fine-tuning gene expression and have been reported relevant for kidney development [19] and PKD [20], [21]. Therefore, it seemed reasonable to suppose they could be involved in orchestrating the observed gene expression changes. We analyzed 47 samples using Agilent microRNA arrays spanning the P12/P14 mutant and control groups (Table S7) and failed to identify differences (fdr p value<0.05). No differences were found by real-time quantitative PCR in a set of 3 microRNA's previously linked to cystic disease (mir15a, mir21 and mir31 [20], [22]; Figure 3).

### Meta-Analysis of Published Gene Array Data of *Pkd1* Wild-Type Tissues Identifies Gene Modules Likely Involved in Kidney Maturation and Cyst Formation

**Figure 4:**
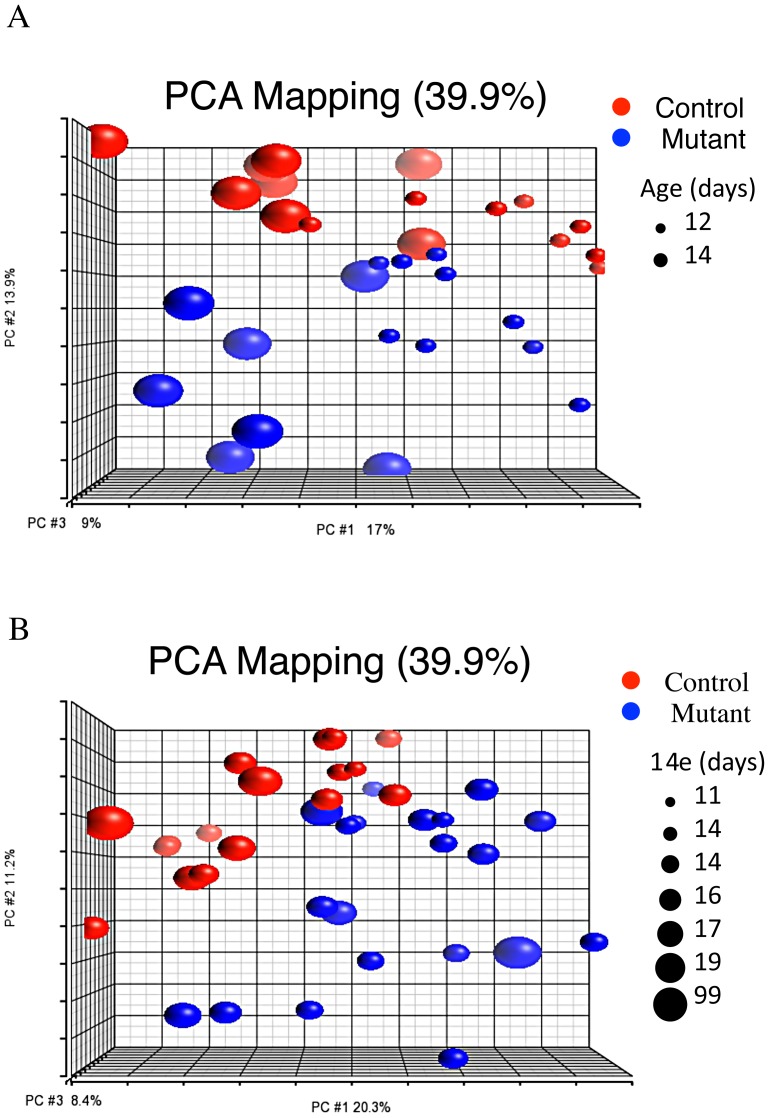
Meta-analysis of 1,114 gene expression arrays identifies gene clusters differentially expressed in *Pkd1* mutant kidneys. PCA plot showing that module 17 separates mutant and control groups along the second principal component in both test (A) and validation (B) groups (meta-analysis genes in Table S9).

If, as our data suggest, the gene expression changes in *Pkd1* mutants are not due to major re-wiring of gene networks, it seems reasonable to suppose that modules of genes normally co-regulated in distinct biological conditions (such as different organs, developmental stages, or activation status of signaling pathways) might also be co-regulated in *Pkd1* mutants. As a consequence, such modules, when enriched for genes differentially expressed in *Pkd1* mutants, could uncover pathways likely disrupted in mutant animals. To test this, 1114 microarray experiments surveying various biological conditions were downloaded from the Gene Expression Omnibus (GEO; http://www.ncbi.nlm.nih.gov/geo/) repository, and analyzed using WGCNA (Table S8). Of the 49 gene modules thus obtained (Table S9), two had significant overlap with the mutant signature set (Modules 5 and 17, p<0.001, Table S10). Alternatively, ranking these modules according to how accurately they predict (mutant x age) status in the test dataset, module 17 had the lowest misclassification rate, clustering only one of the mutants with the controls (and none of the controls with mutants; Figure 4). Consistent with the results described in the previous sections, gene ontology analysis showed enrichment for metabolic pathways (Module 5, p = 1.82E-97; Table S11), cell adhesion and organ morphogenesis (Module 17, p = 1.59E-32 and 3.26E-11; Table S12). Furthermore, enrichment for HNF4α-pathways (nuclear receptor subfamily 1, group H, member 4; p = 3.3E-05) was again observed. Analogous analysis searching for modules with enrichment for genes that change with the normal P12 to P14 transition identifies modules 5 and 17 as the most significant gene clusters (Table S10), further suggesting that similar transcriptional programs are involved in both early stages of cyst formation and postnatal maturation.

### *HNF4α* Is a Modifier of *Pkd1*-Cystogenesis

**Figure 5:**
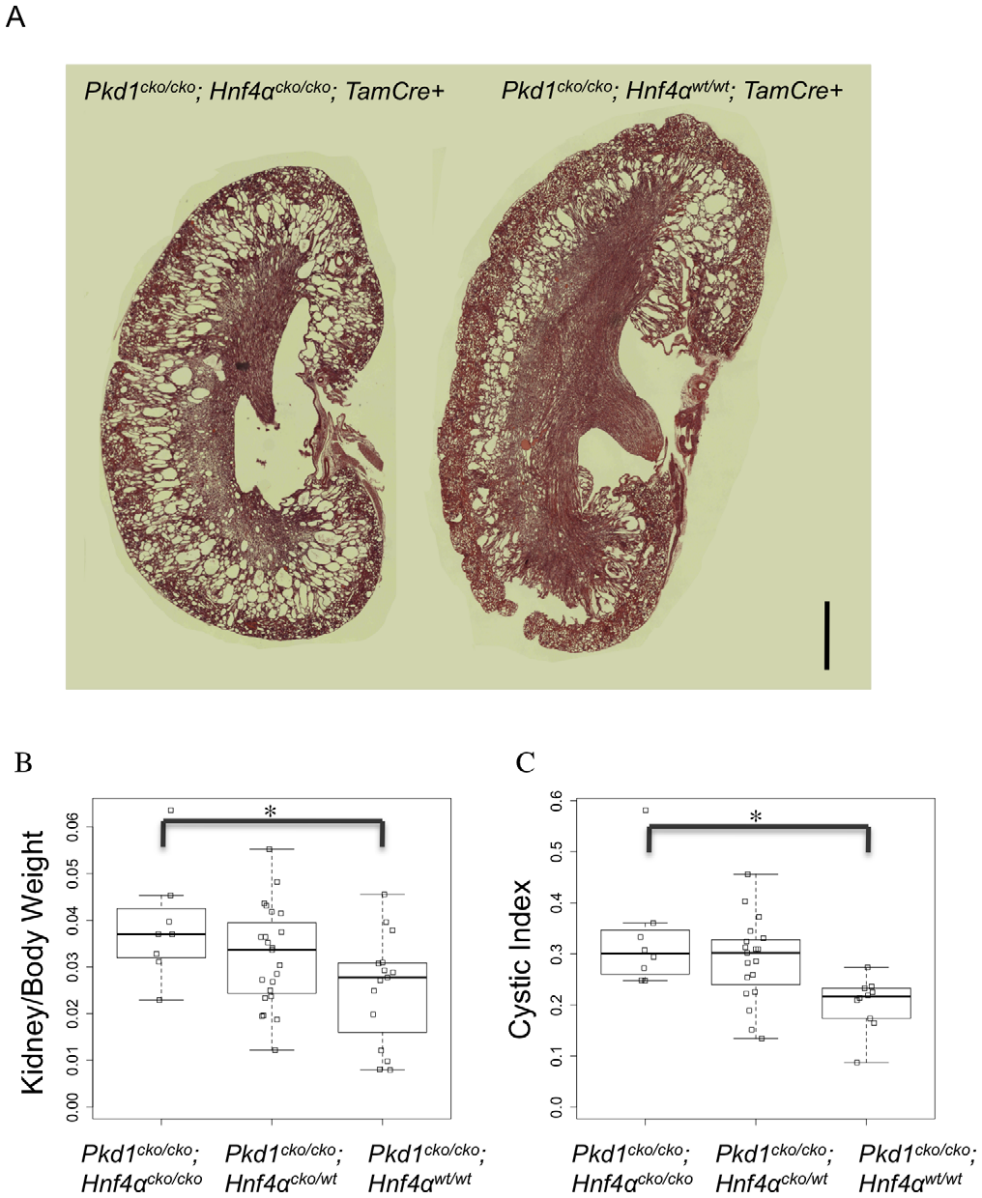
*Hnf4α* is a modifier of *Pkd1*-cystogenesis. (A) Representative kidney histology of *Pkd1^cko/cko^; Hnf4α^cko/cko^; Tamoxifen-Cre+* (left) and *Pkd1^cko/cko^; Hnf4α^wt/wt^; Tamoxifen-Cre+* (H&E stain; scale bar: 1000 mm). (B and C) Boxplot of kidneys/(2*body weight) (B; 95% confidence interval: 0.0021 to 0.0245, p = 0.02271) and cystic index (C; 95% confidence interval: 0.0334 to 0.2304, p = 0.01311; blank squares: samples).

The independent identification of HNF4α as a relevant transcription node using various strategies suggested that it could play a role in *Pkd1*-cystogenesis. In fact, work in our lab had previously shown increased HNF4α activity in *Pkd1* null placentas and kidneys [23]. To further probe the role of metabolic pathways in general, and HNF4α in particular, in *Pkd1*-cystogenesis, *Pkd1^cko^* mice were bred to *Hnf4α^cko^* mice. Gene knockout was induced at P7, and the animals were harvested between P12 and P21. Analyzing kidney/body weight and cystic index between P19 and P21 in a set of 46 cre positive *Pkd1^cko/cko^* animals (8 *Hnf4α^cko/cko^*; 23 *Hnf4α^cko/wt^*; 15 *Hnf4α^wt/wt^*), our results suggest that the presence of HNF4α has a significant protective effect on cyst formation (Figure 5). Despite the variability in phenotype, the 95% confidence interval suggests a reduction of between 5 and 63% in the kidney/body weight (p = 0.023), and between 10 and 67% in cystic index (p = 0.013), an effect similar to that reported with the use of metformin, a metabolism-altering drug [24].

### Urinary Metabolomics Shows Altered Metabolic Profile in Mutant Animals

**Figure 6:**
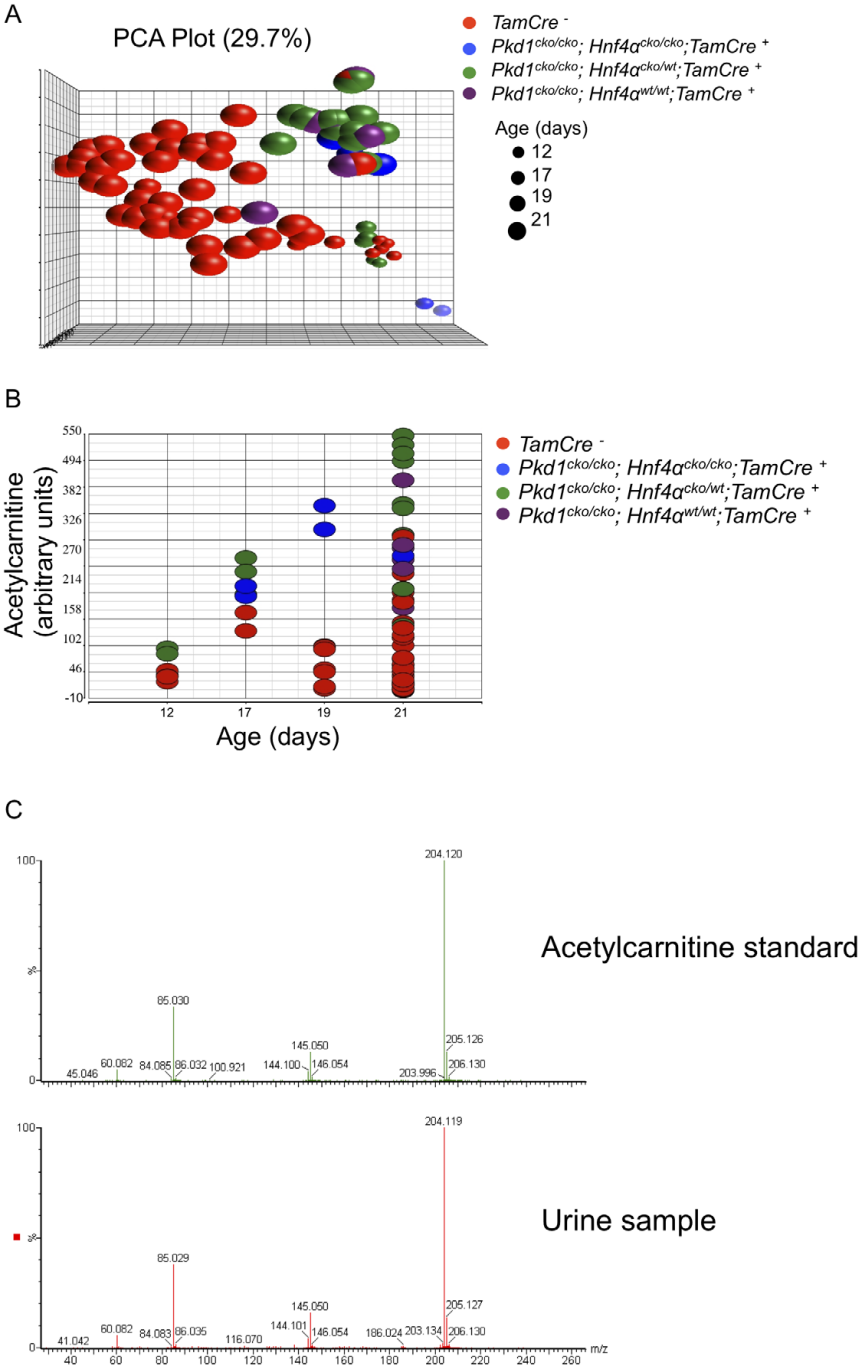
Urinary metabolomics shows distinct patterns in cystic versus control mice. (A) PCA plot of positive mass spectra identified in urine (Table S13), showing separation of control and *Pkd1* mutant samples, irrespective of *Hnf4α* genotype, along the first principal component. (B) Normalized levels of acetylcarnitine in urine samples of *Pkd1/Hnf4α* mice at different ages. (C) MS/MS fragmentation spectra of acetylcarnitine authentic standard versus a representative urine sample.

Metabolic changes in the urine of 84 animals, ranging from P12 to P21, were assessed using ultraperformance liquid chromatography followed by quadrupole time-of-flight mass spectrometry (UPLC/QTOFMS). Principal component analysis suggests that *Pkd1^cko/cko^* cystic mutants have a distinct profile of excreted metabolites, irrespective of *Hnf4α* genotype (Figure 6A). A set of 1458 mass spectra (528 electrospray ionization (ESI) positive, Table S13; 930 ESI negative, Table S14; mean>0.1 in both mutant and control groups, fdr-p<0.02, fold change>2) were differentially detected between control and mutants, 84 of which could be mapped to KEGG ids recognized by IPA (54 of which were unique identifiers in KEGG pathways; Table S15), and were used for further pathway analysis. The results suggest altered activity in several metabolic pathways (Table 1 and Table S16), including purine and tyrosine metabolism. Tyrosine metabolites are known to cause kidney injury, and at least one mouse model of hepatorenal tyrosinemia shows evidence of altered cAMP signaling [25], a pathway thought to be involved in PKD [16], [26], [27].

**Table 1:** Metabolic pathways significantly different in the urine of mutant mice.

Canonical Pathway	p-value	Ratio (Differentially detected compounds/compounds in pathway)
Tyrosine Metabolism	1.14E-02	7/197
Oxidative Phsphorylation	2.76E-02	2/159
Purine Metabolism	4.02E-02	4/391
Aminoacyl-tRNA Biosynthesis	4.30E-02	3/78

Using partial least squares, urinary acetylcarnitine was identified as the metabolite that most accurately predicts mutant status, with levels higher in mutants at all time points (Figure 6B). Verification of acetylcarnitine was obtained by comparing fragmentation patterns of urine samples with authentic standard by tandem mass spectrometry (Figure 6C). Increased concentrations of l-carnitine and acylcarnitines have been described in patients with disorders of organic acid metabolism [28], pathologies that also manifest polycystic kidneys [29]. Taken together, these results are consistent with the observed changes in metabolic pathways playing a role in modulating cystogenesis.

## Discussion

A handful of studies have previously examined gene expression changes in human and mouse renal cystic tissue. While they have reported interesting differences that affect multiple signaling pathways, they have a number of important limitations that greatly reduce their informativeness. In the case of human ADPKD, two studies have described global gene expression changes in *Pkd1*-related cyst formation using cells derived from non-cystic/cystic human kidneys [30], [31]. In one, the investigators performed SAGE analysis of four immortalized cell lines derived from normal and cystic biliary epithelial cells (N = 1 each) and normal and cystic renal epithelial cells (N = 1 each). Top differentially expressed genes (N = 250) were selected for a custom array that was then used to query gene expression in eight cystic kidneys removed from individuals with end stage kidney disease and six undefined “normal” kidneys. Only ∼50% of the genes had detectable signal above background in this set of specimens, and differential expression was detected for 74. The SAGE and chip patterns were discordant for 12 of the 74 genes.

The second study compared gene expression levels in a total of 13 cysts (grouped into three sizes) from five cystic kidneys along with a “minimally” cystic renal cortical specimen from each specimen to gene expression levels in three non-cancerous “normal control” specimens excised from the renal cortex of three nephrectomized kidneys with isolated renal cell carcinoma. The study did not provide any details about the cell-type composition of the cyst (ie. isolated cyst-lining epithelial cells vs. excised cysts which include both epithelial and non-epithelial cell components).

There are a number of other important considerations relevant for both studies: 1) the number of individuals that was studied was small; 2) experimentally relevant details were not provided for the control groups; 3) when using cystic vs. “control” cell lines extracted from ADPKD patients, defining relevant control lines is a major challenge as cysts arise from various nephron segments; 4) differences may be idiosyncratic to individuals or clones and hard to generalize; 5) cysts from end-stage kidneys may have gene expression patterns that are secondary to the cystic stage or uremic state and not necessarily part of *Pkd1*-related pathways.

There also have been two studies of *Pkd1* mutant mouse models. Chen et al. used a hypomorphic model to track changes in gene expression over the course of the first 3.5 weeks of life (four time points, N = 13 pairs) as the kidney progresses from a globally microcystic to a macrocystic stage [32] and Pandey et al. focused on embryonic kidneys, analyzing *Pkd1* null and control kidneys at E14.5 and E17.5 (N = 3 at each time point) [21]. Both studies have methodologic issues that complicate their interpretation. In the case of the Chen study, analysis of the raw data suggests the presence of one outlier (GSM339412) and at least one sample likely with hybridization artifacts (GSM339408; background higher than signal in one channel, and undetected in the other), and it is unclear how/if they affect the reported results. In the case of Pandey et al, most of the analyses were performed using an uncorrected p-value. When we repeated the analyses using their dataset (GSE24532) and corrected for multiple comparisons, very few probes were significantly differentially expressed (File S1). In fact, some of the genes reported as differentially expressed between mutant and control E14.5 animals seemed to have expression values below background, and high detection p-values, suggesting that their fluctuations could be experimental noise, rather than true biological differences. When we re-analyzed the data keeping only probes with detection p-value<0.01 in all samples, the number of differentially expressed genes between mutants and controls at E14.5 or E17.5 was dramatically reduced, and Gene Set Enrichment Analysis failed to run due to the small number of differentially expressed genes.

Our study has a number of unique features that distinguish it from the others. First, we targeted a poorly understood postnatal renal maturation stage that we had previously shown plays a critical role in determining the rate of cyst formation in response to acquired *Pkd1* inactivation. Second, we compared gene expression patterns from both pre-cystic and early cystic specimens and correlated them to transcriptional programs activated during this late stage of kidney maturation. Third, we used a large number of homogeneous samples, thereby minimizing both biological and experimental noise. Importantly, using rigorous correction for multiple comparisons, we identified a modest number of small, but significant, gene expression changes. Finally, we confirmed our findings in a second, large validation set of specimens that covered a wider temporal window. The profile generated using the test group properly clustered the validation set of specimens.

One important goal of our study was to try to identify transcriptional networks responsible for the cystic state. Using WGCNA to examine co-expression networks, we were surprised to find similar architecture in both mutant and controls. We then extended the study to include meta-analysis of 1114 gene expression arrays that sampled a variety of tissues and biological conditions and obtained similar results. In fact, they suggest that some modules of co-regulated genes are preserved across distinct biological conditions, including *Pkd1* inactivation, and that transcriptional regulation of a few of these modules is responsible for a large fraction of the gene expression changes observed in *Pkd1* mutants. They also provide proof of principle that one can mine databases for clusters of co-regulated genes overlapping with small sets of disease-signature genes to uncover relevant pathways. Finally, these results lead us to speculate that PKD pathogenesis might share some characteristics with biological processes such as ageing, in which the compounding of small changes, rather than few, major differences, determines the phenotype. However, as the mechanisms linking these changes to PC1 remain unknown, it is theoretically possible that a major hub downstream of PC is responsible for all subsequent changes.

Complementing the WGCNA approach, we used knowledge-based network tools to identify from the set of mutant signature genes candidate biological networks with altered activity. One of the advantages of a network-based approach to studying disease is that it identifies key molecules at nodes that may play important roles in modulating disease initiation and progression. Interestingly, we found that a number of the nodes had previously been linked to polycystic kidney disease (TNF, AGT, AVP). For example, TNF-αwas reported to trigger cyst formation in both wild-type and *Pkd2^+/−^* kidney organ cultures [14], suggesting a role for inflammatory pathways in cyst growth. The renin-angiotensin system (AGT) has been reported to accelerate disease progression by enhancing fibrosis and promoting cyst growth [15]. The AVP/vasopressin receptor signaling pathway is thought to promote cystic disease by increasing cAMP levels, which then are thought to promote fluid secretion via PKA-dependent chloride-channels and cystic epithelial cell growth via activation of B-Raf/ERK pathway [27]. Two clinical trials are currently underway testing the hypothesis that drugs which modulate the AGT and AVP nodes will slow progression of disease (ClinicalTrials.gov: HALT PKD NCT00283686; TEMPO 3/4 NCT00428948).

In the current study, we focused on HNF4α because we had multiple lines of evidence implicating the protein in *Pkd1* cystogenesis. As noted elsewhere, we had previously reported altered HNF4α activity in *Pkd1* null placentas and kidneys [23]. Our present data are consistent with those observations, showing significant enrichment for genes linked to metabolic pathways in our mutant signature set and in disease-associated eigengenes. Our metabolomics data further support this connection. HNF4α also was identified by IPA as one of the most highly connected nodes with the most direct links. Therefore, we perturbed the network map by inactivating *Hnf4α* in the context of *Pkd1* inactivation and found that HNF4α in fact plays an important modulatory role in the early onset *Pkd1* cyst model. Given that our studies independently identified three nodes previously implicated as important pathway factors in PKD and predicted a fourth that we confirmed with genetic manipulation of the gene, we postulate that some of the other nodes may also be important disease modifiers (ie. nitric oxide synthase 1 (NOS1), superoxide dismutase 2 (SOD2), SHC-transforming protein (SHC1)). It is perhaps relevant that both SOD2 and SHC1 have a role in regulating mitochondrial activity and response to oxidative stress. Further study will be required to test this hypothesis.

HNF4α is a transcription factor with complex, possibly inter-related functions in the regulation of developmental programs, maintenance of organ differentiation and regulation of metabolic pathways. In the developing embryo and fetus, it plays a critical role in epithelial morphogenesis. Homozygous germline inactivation results in early embryonic demise [33]. Bypassing this lethal stage by tetraploid rescue or conditional inactivation strategies, investigators have shown that *Hnf4α* also is essential for liver and colon development [34]–[37]. In post-developmental organs, inactivation of *Hnf4α* has more subtle, functional effects [38], [39]. Post-partum inactivation of *Hnf4α* in the liver results in fatty livers and altered serum levels of cholesterol, triglycerides, and bile acids whereas inactivation in the colon results in an intact organ with increased susceptibility to injury. Possibly linked to both its developmental and post-developmental roles is HNF4*α*'s key function as a regulator of several metabolic pathways [40], [41]. It may act as a nutritional sensor [36], [40]–[42] with its function regulated mainly by posttranslational modifications and binding to co-regulators [42]. The possible inter-dependence of the different roles for HNF4*α* are in part suggested by studies that show that some of the pathologic consequences resulting from its inactivation appear to be non-cell autonomous. Whereas *Hnf4α* null hepatocytes *in vivo* lack normal polarity and epithelialization, cultured *Hnf4α* null cells polarize properly and are indistinguishable from control cells, suggesting that polarity defects might be secondary to abnormal expression of metabolic genes [43].

The role of HNF4α in the kidney is incompletely defined. It is expressed widely in the developing organ and is thought to help regulate mesenchymal to epithelial conversion (MEC) during nephrogenesis [44]. In the adult, *Hnf4α* is expressed mostly in the cortex, and in proximal tubules, with very low levels of expression in collecting ducts [45]. While no comprehensive analysis of post-natal inactivation of *Hnf4α* has been reported, in our pilot studies of *Pkd1* wild type mice we saw no obvious histologic effects of *Hnf4α* inactivation in the kidney during the timeframe used in this study (unpublished observation). In contrast, we found that inactivation of *Hnf4α* in the context of simultaneous *Pkd1* inactivation significantly worsened the cystic phenotype. Therefore, we speculate that the paucity of cortical cysts in the early stages of cyst formation, and the increased severity of cystic disease in double *Hnf4α/Pkd1* knockout might reflect a protective role of HNF4α. In this scenario, HNF4α activity could be compensatory to *Pkd1* inactivation. Alternatively, HNF4αcould be a marker (or driver) of broader metabolic changes, possibly even in other organs (liver), and it is possible that the relevant modifier is the metabolic context, rather than one specific metabolic regulator. According to this model, HNF4α-related changes in metabolic and/or other signaling pathways could change the way cells respond to signals, conferring context-dependent differences in the susceptibility to the effects of *Pkd1* inactivation. However, it has to be noted that animals induced after P14 do eventually get cystic (late-onset model), suggesting that changing metabolic contexts may retard, but is unlikely to prevent, disease progression. It also remains to be shown if the same networks/pathways are involved in early- and late-onset *Pkd1* disease models.

Urinary metabolomic studies provide additional evidence indicating that dysregulated metabolic pathways may be a property common to multiple forms of PKD. In the current study, we found metabolites of four metabolic pathways significantly different in the urine of early and moderately cystic *Pkd1* mutant mice (Table 1). Taylor et al analyzed the urine of female control and minimally cystic *jck* mice, a mouse model for human nephronophthisis that has a mutation in the murine orthologue of human *NPHP9*, and found seven metabolic pathways that differed significantly between genotypes [46]. Abbiss et al compared the urine of control and moderately cystic Lewis autosomal recessive polycystic kidney disease rats and found altered levels of 2-ketoglutaric acid, allantoin, uric acid and hippuric acid [47].

Together, these data suggest that metabolic pathways may play an important role in PKD pathobiology. The link between metabolic changes and cell behavior is well recognized in cancer cells, where glycolysis in the presence of oxygen (Warburg effect) and activation of synthesis of substrates for cellular components have long been noticed [48]. In the case of PKD, interpretation of the metabolic differences is more complicated. In late stages of the disease, modest differences in proliferation rate and uremic status may be a confounding factor. However, the transcriptional changes and some of the metabolic differences are observed in minimally cystic animals when proliferation rates are either normal or minimally different. Moreover, the urine samples of moderately cystic mutants in our dataset do not cluster by PCA with those of younger normal mice who have a much higher proliferation rate. In sum, it is unlikely that changes in proliferation rates alone can explain the differences in metabolism. These changes in metabolism may be upstream (or downstream) of several pathways, such as the AMP/ATP-sensing AMP-kinase, or aminoacid-activated mTOR signaling, previously linked to PKD [49]. Our results suggest an alternative explanation for metabolic changes, based on differences in HNF4α activity.

An important question prompted by these observations is whether the observed metabolic effects are the result of the cystic state or a causal factor in promoting cyst growth. We think the data suggest that both answers are true. As noted above, the cysts form contemporaneous with the changes in gene expression, and the altered cellular properties associated with the cystic state likely result in altered cellular metabolism. In this respect, the altered metabolic profile and gene expression patterns may be a consequence of cystic transformation. However, we also show that manipulating *Hnf4α* activity, and thereby presumably altering the regulation of metabolic gene expression, also modifies the phenotype. This observation suggests that metabolic pathways may be a disease modifier. Future studies will be necessary to determine whether it is the altered activity of metabolic pathways per se or some other functional consequence of *Hnf4α* activity that is responsible for our findings. It also will be important to determine the most proximal, direct, link between *Pkd1* deletion and changes in metabolic pathways.

We have previously shown that proliferation rates drop normally in mutant kidneys induced at P12, and suggested that, assuming proliferation can be used as a marker of developmental status, it was unlikely that *Pkd1* deletion induced developmental arrest [4]. Here we show that changes in gene co-expression networks correlate with kidney maturation between P12 and P14 in control animals. Furthermore, we observe very similar network changes in mutant animals during the same interval. WGCNA analysis seems therefore sensitive enough to detect changes in developmental status, and the results imply that mutant kidneys are neither developmentally arrested, nor de-differentiated. It is however likely, given our GO results, that genes normally involved in developmental processes are differentially expressed in mutant kidneys.

Several groups have previously implicated microRNAs in PKD pathogenesis. Using both a comprehensive screening method (microarray) on a large number of specimens and targeted real-time quantitative PCR of a small number of PKD-associated microRNAs on a subset of specimens, we failed to identify significant differences in microRNA expression during the early stages of cyst formation. Several factors could account for these discrepancies: 1) method sensitivity; 2) differentially expressed microRNA's could be cell/model/disease stage-specific; 3) asynchronous waves of microRNA changes may occur in different nephron segments. It is interesting to note, however, that conditional knockout of Dicer in proximal tubules, a nephron segment that becomes cystic when *Pkd1* is inactivated, does not result in cystic disease in mice up to 6 months of age, despite microRNA depletion [50] (Dr. Dong, personal communication).

Genes in both the mutant-signature and ME2 module are significantly enriched in gene ontology categories involved in development, which likely reflect the remodeling of nephron segments (anatomical structure development, p = 3.42E-09; cell adhesion, p = 7.87E-09) and surrounding vasculature (vasculature development, p = 1.13E-05). This suggests that additional significant categories such as cell differentiation (p = 9.31E-08) could be related to morphological changes, rather than due to developmental arrest/dedifferentiation, consistent with our results of normal kidney maturation and preservation of gene correlation networks in mutants. Enrichment for metabolic pathways, though also seen in the ME2 cluster (heterocycle metabolic process, p = 3.13E-06; regulation of steroid metabolic process, p = 3.32E-03), is particularly noticeable in the turquoise module, which includes oxidation/reduction (p = 1.16E-14), protein metabolic process (p = 9.12E-09) and glucose metabolism (1.58E-08). As this module seems to be correlated with kidney maturation and possibly to the susceptibility to the effects of *Pkd1* deletion, our data highlight the metabolic context as a likely modifier of cystogenesis, a hypothesis further supported by the observed genetic interaction with *Hnf4α*. It would be interesting to see if perturbing the metabolic context could turn the early-onset into a late-onset model in animals induced before P12. It also remains to be shown that metabolic changes play a role in the late onset form of the disease.

In conclusion, the present comprehensive systems biology approach provided important insights into PKD biology and kidney maturation. The data suggest that *Pkd1*-cystogenesis does not require/cause developmental arrest, and occurs in the context of gene networks essentially similar to those that regulate/maintain normal kidney morphology/function. We also propose that postnatal kidney maturation is accompanied by changes in metabolic pathways that are likely to be modifiers of *Pkd1*-cystogenesis and could underlie the differences in the kinetics of cyst formation in early- or late-onset disease models. Finally, we show that *Hnf4α* is a modifier of *Pkd1*-cystogenesis.

## Methods

### Ethics Statement

All studies were performed using protocols approved by the JHU and NIH Animal Care and Use Committee, and mice were kept and cared in pathogen-free animal facilities accredited by the American Association for the Accreditation of Laboratory Animal Care and meet federal (NIH) guidelines for the humane and appropriate care of laboratory animal.

### Animals

Fifth-generation C57/BL6 *Pkd1^cko^* mice were crossed to fifth-generation C57/BL6 tamoxifen-Cre (B6.Cg-Tg(Cre/Esr1)5Amc/J mice (stock 004682), Jackson Laboratories) and C57/BL6 congenic B6.129S4-*Gt(ROSA)26Sor^tm1Sor^*/J (stock 003474, Jackson Laboratories) to produce the mice used in these studies. The *Pkd1^cko^* thus obtained were bred to *Hnf4α^cko^* mice (B6.129X1(FVB)-*Hnf4α^tm1.1Gonz^*/J (stock 004665, Jackson Laboratories). Cre recombinase activity was induced by intraperitoneal injection of tamoxifen (10 mg/40 g) in corn oil (Sigma-Aldrich) in nursing moms of mice<10 days of age. Mice were euthanized by isofluorane treatment followed by cervical dislocation.

### Histology and Cystic Index

Specimens were fixed in 4% paraformaldehyde buffered solution, pH 7.4 at 4°C overnight, washed in PBS for 2 h, dehydrated in 50% ethanol for 2 h and stored in 70% ethanol at room temperature. The kidneys were cut in half along the longitudinal axis, embedded in paraffin and sectioned in 5-µm slices for hematoxylin and eosin staining. To calculate the cystic index, a composite image of a longitudinal midline kidney section was assembled using Axiovision (Zeiss), and the total kidney and cystic areas were measured using ImageJ [51]. Statistical analysis was performed using Student's t-test.

### mRNA and microRNA Profiling

Total RNA was isolated from kidneys using Trizol reagent (Invitrogen). For mRNA studies, a subsequent step of RNA isolation using RNeasy plus kit was performed (Qiagen). Illumina's MouseRef-8 v1.0 or v2.0 BeadChip expression arrays with sample processing performed by the Johns Hopkins Bayview Medical Campus Genomics Core. For microRNA expession profiling, Agilent miRNA arrays were used with the Johns Hopkins Medical Institute microarray core (http://www.microarray.jhmi.edu).

microRNA real time PCR amplification was performed using TaqMan MicroRNA reverse transcription kit (4366596, Applied Biosystems), and the TaqMan microRNA assays has-miR-21, mmu-miR-31, has-miR-15a, normalized to microRNA snoRNA 202 control (respectively, RT 397, RT 185, RT 389, RT 1232; Applied Biosystems).

### Microarray Data Analysis

Gene expression data were normalized using software provided by the Bioconductor project [52]. Briefly, we imported gene expression data using the lumi package, followed by preprocessing using variance stabilization transformation and quantile normalization [53], [54]. The data were filtered to include only probes with detection call p value<0.05 in at least one of the arrays and COMBAT software employed to remove batch effects [55]. When combining datasets of the two different Illumina array versions (v1.0 and v2.0), only probes present in both versions were included. For the microRNA data, quantile-normalization was applied. For both mRNA and microRNA normalized data, Partek was used to obtain principal component analysis and heatmap plots, and to identify differentially expressed probes/genes using ANOVA-based methods and fdr multiple comparison correction. For meta-analysis of published gene expression data, CEL files of experiments performed using Affymetrix Mouse Genome 430 2.0 arrays (platform GPL1261) were downloaded from GEO (http://www.wip.ncbi.nlm.nih.gov/geo/), imported into Partek using RMA, followed by quantile normalization. All microarray data have been uploaded to the GEO database (Accession GSE32586).

### Weighted Gene Co-Expression Network Analysis

For weighted gene co-expression network analysis (WGCNA) [56], the WGCNA R package was used [11]. Briefly, soft thresholding powers were chosen based on the approximate scale-free topology criterion [56], and applied blockwise network construction and module detection to the normalized expression matrix. We then correlated the modules with age and/or mutant status.

For comparative network analysis, multiSetMEs [11] was used to identify consensus modules for the 4 experimental groups: P12-mutant, P12-control, P14-mutant and P14-control, and to determine module preservation and quantify eigengene correlations [17].

### Pathway Analysis

Genomatix Software Suite (Genomatix Inc.) was used to identify gene ontology, and pathway enrichment. To build knowledge-based networks, Ingenuity Pathway Analysis was used (IPA; Ingenuity Systems). Briefly, matrices were generated with fold-change and q-value for each of the comparisons of interest (i.e. age and mutant status) and considered genes with more than 1.2 fold change and q<0.05 as network eligible molecules. IPA was then used to screen the Ingenuity Knowledge Base (January 2011 release) for reported interactions involving these genes, to identify networks that maximize connectivity and to score them based on the number of network eligible molecules they contain. The network score corresponds roughly to –log10 (p value).

### Urinary Metabolomics

Urine samples were collected at the time of euthanasia, diluted with 20 volumes of water and spun at 14,000× g for 10 min at 4C. The supernatant was transferred to an autosampler tube and analyzed by ultraperformance liquid chromatography coupled with electrospray ionization quadrupole time-of-flight mass spectrometry. MarkerLynx software was used to align and deconvolute the mass chromatographic data and to generate a data matrix consisting of peak areas corresponding to a unique m/z and retention time. Statistical analysis was carried out using Partek, employing unpaired t-test comparing cre positive and negative animals, and partial least square (PLS) analysis. The relevant m/z to KEGG ids [57] were identified by searching the Scripps Center for Metabolomics Database with 5 ppm accuracy and possible modifications (+H, +NH4, +Na, +H-2H2O, +H-H2O in the positive mode; −H, −H2O-H, +Na-2H, +Cl, +K-2H in the negative mode) [58]. Verification of acetylcarnitine (m/z 204.123) was obtained by comparing fragmentation patterns of urine samples with authentic standard by tandem mass spectrometry. For generation of MS/MS fragments, the mass spectrometer collision energy was ramped from 6 to 40 V. For pathway enrichment analysis, Ingenuity Pathway Analysis was used.

## Supporting Information

File S1 Re-analysis of GSE24532 published dataset.
(PDF)

Table S1 List of genes in mutant-signature.
(XLS)

Table S2 Gene Ontology Biological Processes classification of genes in mutant-signature.
(XLS)

Table S3 Genes in ME2 module.
(XLS)

Table S4 Gene Ontology Biological Processes classification of genes in ME2 cluster.
(XLS)

Table S5 Genes in Turquoise module.
(XLS)

Table S6 Gene Ontology Biological Processes classification of genes in Turquoise module.
(XLS)

Table S7 Quantile-normalized microRNA expression data.
(XLS)

Table S8 GEO datasets used for meta-analysis.
(XLS)

Table S9 Module membership of genes in meta-analysis.
(XLS)

Table S10 Gene modules identified in meta-analysis containing genes in either the mutant-signature or that normally change between P12 and P14 (age switch).
(XLS)

Table S11 Gene Ontology Biological Processes classification of genes in Module 5 of the meta-analysis.
(XLS)

Table S12 Gene Ontology Biological Processes classification of genes in Module 17 of the meta-analysis.
(XLS)

Table S13 Differentially detected mass spectra in the positive electrospray ionization mode.
(XLS)

Table S14 Differentially detected mass spectra in the negative electrospray ionization mode.
(XLS)

Table S15 Differentially detected mass spectra with putative KEGG ids in KEGG Pathways.
(XLS)

Table S16 Differentially detected metabolites in each of the significant pathways.
(XLS)
