# Supplementary material for: Network Analysis of a Pkd1-Mouse Model of Autosomal Dominant Polycystic Kidney Disease Identifies HNF4α as a Disease Modifier
Source: PLoS Genet. 2012 Nov 29;8(11):e1003053. doi: 10.1371/journal.pgen.1003053 (PMC3510057; doi:10.1371/journal.pgen.1003053)
Supplement: File S1 — Re-analysis of GSE24532 published dataset. (PDF) [file pgen.1003053.s001.pdf]

To verify the results reported by Pandey et al. [1], we downloaded the dataset (GSE24532) from GEO, imported into R, log2-transformed, quantile normalized, and analyzed using limma package with empirical Bayes methods, comparing mutant vs. controls at E.14.5 and E17.5, and E17.5 vs. E14.5 within the mutant and control groups, as described [1]. In their report, genes were considered differentially expressed in the mutant vs. control comparisons if they had >2-fold change and unadjusted p-value<0.05. As shown in table 1, using these criteria we obtained similar numbers of differentially expressed genes (the higher number in our analysis could reflect genes represented by multiple probes). However, when multiple comparisons corrections were applied, very few probes were significantly differentially expressed (table 1). In fact, some of the genes reported as differentially expressed between mutant and control E14.5 animals, when unadjusted p-values were used, seemed to have expression values below background, and high detection p-values, suggesting that their fluctuations could be experimental noise, rather than true biological differences (table 2), though we could not be completely sure as to which probes were being mapped to each of the gene symbols.

We therefore re-analyzed the data keeping only probes with detection p-value<0.01 in all samples, and collapsed probe values to the probe with highest mean value, to avoid inflating the significance of GSEA categories due to replicate probes of the same gene. Using limma and correcting for multiple corrections, the number of differentially expressed genes was then dramatically reduced (table 3), and GSEA failed to run due to small number of genes (E14.5 or E17.5 mutant vs. control).

Table 1. Number of differentially detected genes (unadjusted p-value and 2-fold change).

	Down regulated		Unchanged		Up regulated	
	unadjusted p-value	BH-adjusted p-value	unadjusted p-value	BH-adjusted p-value	unadjusted p-value	BH-adjusted p-value
E14.5 Mutant vs. Control	290	3	30110	30677	282	2
E17.5 Mutant vs. Control	446	18	29571	30648	665	16
E17.5 Mutant vs.E14.5 Mutant	1723	1305	26820	27659	2139	1718
E17.5 Control vs.E14.5 Control	1992	1452	26756	27657	1934	1573

Table 2. Lowest detection p-value among all samples of reported differentially expressed genes between mutants and controls at E14.5

ID	Transcript	Symbol	Maximum Expression Value	Lowest DetectionPvalue
ILMN_2750053	ILMN_222475	Ptprj	6.60	0.2415
ILMN_2808191	ILMN_240635	Ddo	14.46	0.1314
ILMN_2887824	ILMN_215082	Cars	15.90	0.1250
ILMN_1216885	ILMN_211881	P2rx7	-1.86	0.4744
ILMN_2607675	ILMN_210638	H2-Ab1	629.97	0.0000
ILMN_3006451	ILMN_238311	Jak3	49.05	0.0053
ILMN_2655721	ILMN_194854	Stat1	448.13	0.0000
ILMN_2449157	ILMN_188021	Tnfsf9	35.69	0.0299
ILMN_2671320	ILMN_216459	Il23a	10.70	0.1325
ILMN_1255750	ILMN_204884	Il7r	-6.11	0.5940
ILMN_3000090	ILMN_222952	Piwil4	2.66	0.2927
ILMN_1233469	ILMN_202525	Ldlr	108.94	0.0000
ILMN_1223179	ILMN_196750	H2-T23	180.17	0.0000
ILMN_2990661	ILMN_218140	Pnliprp2	12.51	0.1645
ILMN_2656864	ILMN_193838	Chat	6.80	0.2382
ILMN_1245963	ILMN_205144	Ache	1.37	0.3739
ILMN_2633412	ILMN_210564	Chdh	5.62	0.2532
ILMN_2704285	ILMN_216798	Pklr	24.99	0.0673
ILMN_1249371	ILMN_219423	B3gat1	6.07	0.2179
ILMN_2416670	ILMN_184200	Ttn	38.18	0.0246
ILMN_1225605	ILMN_220227	Pigr	17.97	0.1143
ILMN_2590766	ILMN_208915	Fgfr3	7.04	0.2169
ILMN_2573761	ILMN_205595	Map3k4	-1.50	0.4637
ILMN_1223542	ILMN_191923	Zap70	-5.56	0.5481
ILMN_1236465	ILMN_216016	F2rl2	11.48	0.1592
ILMN_2943387	ILMN_190087	Vip	442.81	0.0000
ILMN_1221640	ILMN_212083	Gabrp	19.08	0.0919
ILMN_2752064	ILMN_222595	Htr1d	20.68	0.1026
ILMN_3096212	ILMN_215206	Mgat5	6.14	0.2361
ILMN_2796353	ILMN_240727	Rad23b	1106.54	0.0000
ILMN_2637965	ILMN_213529	Olfr1055	5.89	0.2372
ILMN_2682548	ILMN_217378	Olfr270	10.11	0.1709
ILMN_2798097	ILMN_219789	Olfr549	-4.75	0.5919
ILMN_2690677	ILMN_186554	Clca3	10.99	0.1271
ILMN_2825410	ILMN_256309	Olfr600	0.19	0.3611
ILMN_1213096	ILMN_219876	Olfr1417	5.33	0.2650
ILMN_1252706	ILMN_219714	Olfr702	24.07	0.0748
ILMN_2637389	ILMN_213474	Olfr810	20.84	0.0609
ILMN_2665697	ILMN_199409	Olfr491	14.94	0.1528
ILMN_2844418	ILMN_227331	Olfr1437	18.60	0.1047
ILMN_2934857	ILMN_234756	Olfr591	8.52	0.2030
ILMN_1235408	ILMN_199401	Olfr330	9.75	0.1816
ILMN_2879910	ILMN_220145	Atp6v1b1	71.88	0.0011
ILMN_1236930	ILMN_186723	Trp73	10.32	0.1720
ILMN_2795317	ILMN_184466	Ugt1a6a	477.94	0.0000
ILMN_2994373	ILMN_187828	Ucp1	10.62	0.1709
ILMN_2669801	ILMN_216327	Nt5c1b	5.36	0.2340
ILMN_2626453	ILMN_192296	Amy1	32.62	0.0449
ILMN_2751094	ILMN_214536	Cyp27b1	487.96	0.0000

ILMN_3113787	ILMN_250769	Grap2	27.16	0.0459
ILMN_3160931	ILMN_241597	Tas2r144	10.40	0.1731
ILMN_2707921	ILMN_219372	Aire	92.72	0.0000
ILMN_2775793	ILMN_224160	Cer1	7.87	0.2094
ILMN_2703082	ILMN_219011	Chd8	2098.02	0.0000
ILMN_2815337	ILMN_215158	Hrg	11.91	0.1592
ILMN_2936769	ILMN_251740	1700061G19Rik	7.61	0.2297
ILMN_3163577	ILMN_255013	Scn3b	7.97	0.2062
ILMN_2610223	ILMN_210889	Sox5	10.41	0.1752
ILMN_1238008	ILMN_201662	Wiz	44.98	0.0064
ILMN_2869715	ILMN_219122	Nell2	11.37	0.1699
ILMN_1246734	ILMN_221338	Osbp2	19.66	0.1026
ILMN_2551988	ILMN_202749	Adam15	11.20	0.1741
ILMN_3156714	ILMN_261324	Astn2	19.24	0.1058
ILMN_1218111	ILMN_193844	4930503B20Rik	-7.48	0.6485
ILMN_2759687	ILMN_223128	Hlf	6.76	0.2190
ILMN_1256787	ILMN_212448	1700019L03Rik	18.82	0.0855
ILMN_2907915	ILMN_258440	BC049730	-8.72	0.7062
ILMN_2750201	ILMN_222484	1700023I07Rik	4.28	0.2682
ILMN_2852672	ILMN_218079	Tspan32	12.41	0.1549
ILMN_2881498	ILMN_191283	Tsnaxip1	7.09	0.2212
ILMN_2597124	ILMN_209577	Lnx1	16.86	0.1143
ILMN_2488888	ILMN_192524	2310061G22Rik	15.85	0.1250
ILMN_2982288	ILMN_210151	Rnf133	8.42	0.2051
ILMN_2687408	ILMN_217773	Serpinb3c	8.48	0.2051
ILMN_3131679	ILMN_186258	Usp18	277.67	0.0000
ILMN_1221737	ILMN_208758	Crygc	3.21	0.2981
ILMN_2520174	ILMN_195943	6530402F18Rik	9.59	0.1838
ILMN_2454393	ILMN_188637	Ypel1	670.62	0.0000
ILMN_2428117	ILMN_185566	Zcchc2	13.25	0.1474
ILMN_2601155	ILMN_209993	Frzb	290.45	0.0000
ILMN_2760183	ILMN_223167	Ppef2	7.95	0.2094
ILMN_2874193	ILMN_241050	Slc22a14	12.50	0.1506
ILMN_1221784	ILMN_223923	Dio3	8.94	0.1955
ILMN_2456526	ILMN_188879	a	13.23	0.1335
ILMN_1233345	ILMN_199439	Mrgprx1	43.81	0.0128
ILMN_2624100	ILMN_209850	Irf1	-1.71	0.4135
ILMN_2825866	ILMN_232966	Dnm3os	12.78	0.1549
ILMN_1220054	ILMN_217940	Diras1	14.90	0.1389
ILMN_3163163	ILMN_237057	A430078G23Rik	9.22	0.1966
ILMN_2737200	ILMN_221557	Mbp	15.40	0.1036
ILMN_2602789	ILMN_210155	Atp11a	1.09	0.3451
ILMN_1251905	ILMN_205407	E130018O15Rik	18.71	0.1047
ILMN_2914456	ILMN_213600	Plekha5	251.82	0.0000
ILMN_2703598	ILMN_188593	Star	5.55	0.2500
ILMN_2735118	ILMN_221403	Pprc1	889.15	0.0000
ILMN_2436168	ILMN_186514	5330439K02Rik	18.30	0.0801
ILMN_2675812	ILMN_216826	Phox2b	10.23	0.1421
ILMN_2841721	ILMN_187235	Ttc3	18674.16	0.0000
ILMN_2608944	ILMN_210766	1700025H01Rik	3.59	0.2788
ILMN_2509817	ILMN_194810	Atp8a1	144.94	0.0000
ILMN_2735995	ILMN_221466	Samd4	2.98	0.2938
ILMN_3058793	ILMN_251837	Qrich2	-6.17	0.6026

ILMN_2565934	ILMN_204562	Wwox	10.72	0.1656
ILMN_1239219	ILMN_216138	Mx2	76.19	0.0000
ILMN_1248018	ILMN_223378	Foxn4	14.05	0.1197
ILMN_2736976	ILMN_221542	1700093K21Rik	29.03	0.0385
ILMN_2890069	ILMN_235370	Tmprss11a	19.20	0.0566
ILMN_2551612	ILMN_202700	8430426J06Rik	21.48	0.0908
ILMN_2617858	ILMN_211630	Ovol1	513.74	0.0000
ILMN_3105499	ILMN_245521	Dlgap4	517.19	0.0000
ILMN_2827515	ILMN_255891	EG436332	10.21	0.1752
ILMN_1249991	ILMN_220519	Cd96	9.05	0.1891
ILMN_2690968	ILMN_210338	Rnf139	134.35	0.0000
ILMN_1234267	ILMN_214213	Pkhd1l1	30.53	0.0449
ILMN_1251725	ILMN_210690	Ly6g6c	54.44	0.0021
ILMN_2714480	ILMN_219858	Dsg1a	2.10	0.3515
ILMN_1248909	ILMN_219695	Itih5	47.07	0.0107
ILMN_2627744	ILMN_212553	Cyp8b1	37.50	0.0267
ILMN_3004786	ILMN_214238	Ccdc39	11.59	0.1731
ILMN_1239070	ILMN_186876	Myo18b	-6.96	0.6239
ILMN_2891245	ILMN_218398	Rhbdl2	6040.24	0.0000
ILMN_2497831	ILMN_193507	Proz	10.59	0.1763
ILMN_2819311	ILMN_258864	Tdpoz2	13.60	0.1506
ILMN_2926202	ILMN_191877	V1ri2	22.12	0.0545
ILMN_1260161	ILMN_213370	Mapk15	17.30	0.0865
ILMN_1227309	ILMN_211910	D18Ertd653e	-5.21	0.5321
ILMN_1213174	ILMN_203841	A330017A19Rik	96.60	0.0000
ILMN_2505392	ILMN_194323	4921513D23Rik	1480.48	0.0000
ILMN_1255629	ILMN_210734	E130309F12Rik	77.27	0.0000
ILMN_2811775	ILMN_225536	4930428D18Rik	6.52	0.2276
ILMN_1254295	ILMN_220261	Sox21	32.39	0.0342
ILMN_3009928	ILMN_242965	4930524E20Rik	12.37	0.1528
ILMN_2848214	ILMN_219907	Npsr1	29.51	0.0438
ILMN_2780286	ILMN_249967	Scube3	187.17	0.0000
ILMN_2919325	ILMN_221668	Serpinb9c	14.26	0.1335
ILMN_2426105	ILMN_185326	6530401N04Rik	11.24	0.1795
ILMN_2495446	ILMN_193252	0610037M15Rik	86.54	0.0000
ILMN_3017818	ILMN_257943	9030619P08Rik	-3.65	0.5246
ILMN_2873131	ILMN_232794	Trp53i11	100.91	0.0000
ILMN_2661820	ILMN_215633	Agxt2l1	45.21	0.0150
ILMN_3017615	ILMN_231586	Syne1	8.49	0.2019
ILMN_2749223	ILMN_222413	1600014C23Rik	30.81	0.0449
ILMN_2724583	ILMN_220626	Otog	16.78	0.1303
ILMN_1226696	ILMN_220733	Rab39	15.08	0.1314
ILMN_1217878	ILMN_188023	4833409A17Rik	25.68	0.0395
ILMN_2989312	ILMN_210626	Gdap1l1	52.42	0.0085
ILMN_2900431	ILMN_223173	Rorc	111.14	0.0000
ILMN_2735829	ILMN_189625	Kiss1	63.52	0.0011
ILMN_2604307	ILMN_210308	Havcr1	19.18	0.0620
ILMN_1231476	ILMN_213249	Aspn	16.62	0.1239
ILMN_2634888	ILMN_213225	Cryaa	17.99	0.1143
ILMN_2609813	ILMN_210847	Chi3l1	24.74	0.0662
ILMN_1241923	ILMN_213348	Msh5	3.20	0.2831
ILMN_1227845	ILMN_213193	BC018465	46.14	0.0096
ILMN_2647218	ILMN_212092	Sez6l2	2.62	0.2970

ILMN_2942492	ILMN_213683	Rbm5	6382.54	0.0000
ILMN_1259168	ILMN_193464	6330407A03Rik	71.06	0.0000
ILMN_3128363	ILMN_245991	Slc14a2	50.25	0.0085
ILMN_2505476	ILMN_194331	Prom1	10.81	0.1806
ILMN_2700292	ILMN_218792	H13	1033.21	0.0000
ILMN_1217280	ILMN_221412	4930528F23Rik	25.13	0.0748
ILMN_1254036	ILMN_188761	Disp2	-9.84	0.7083
ILMN_2830860	ILMN_211717	Gadd45gip1	49.02	0.0075
ILMN_2903889	ILMN_220548	BC066107	195.42	0.0000
ILMN_2634501	ILMN_213181	Rab27b	-2.29	0.4359
ILMN_2731760	ILMN_221163	Myo1f	44.18	0.0171
ILMN_1222677	ILMN_194306	Hoxb13	18.22	0.1047
ILMN_3139800	ILMN_249242	Bre	680.12	0.0000
ILMN_2650502	ILMN_214662	Gdap10	64.48	0.0032
ILMN_2822359	ILMN_214039	Svs2	12.75	0.1496
ILMN_1224664	ILMN_211060	Fbxl11	1154.02	0.0000
ILMN_1216437	ILMN_218945	Rtn3	1600.84	0.0000
ILMN_3102736	ILMN_243152	Gm129	144.29	0.0000
ILMN_2660048	ILMN_215480	Abcc10	27.79	0.0342
ILMN_2918499	ILMN_212008	Abcb1b	18.62	0.1154
ILMN_2664660	ILMN_188455	Aldh5a1	992.67	0.0000
ILMN_2924677	ILMN_209494	Kcnj1	199.69	0.0000
ILMN_2642339	ILMN_194107	Slc1a2	6.64	0.2147
ILMN_2633996	ILMN_213132	Epha1	23.25	0.0791
ILMN_2653871	ILMN_214955	Scd4	16.93	0.0897
ILMN_1242601	ILMN_208701	Grm5	-12.99	0.9209
ILMN_2426965	ILMN_185428	Tpm3	11.06	0.1688
ILMN_2734729	ILMN_221372	H2-Aa	80.60	0.0000
ILMN_1217041	ILMN_222000	Cd86	27.17	0.0556
ILMN_1246921	ILMN_205462	Bub3	15.38	0.1485
ILMN_2655677	ILMN_212852	Mdh1	1824.91	0.0000
ILMN_3112268	ILMN_215821	Dnmt3l	24.59	0.0716
ILMN_2461299	ILMN_189442	Cxcl16	-5.67	0.5502
ILMN_2469578	ILMN_190370	Fmo2	-0.84	0.4509
ILMN_2704777	ILMN_213983	Cyp2d26	2194.77	0.0000
ILMN_2775030	ILMN_224114	Folr2	46.46	0.0053
ILMN_2588139	ILMN_208622	H2-K1	406.86	0.0000
ILMN_1248382	ILMN_196741	H2-D1	919.32	0.0000
ILMN_1221298	ILMN_192350	Tuba8	15.09	0.1079
ILMN_2682574	ILMN_217381	Pla2g5	17.27	0.1314
ILMN_1213632	ILMN_195958	Pck1	118.61	0.0000
ILMN_2999670	ILMN_221105	St8sia5	34.42	0.0321
ILMN_2646260	ILMN_214276	Inpp4a	9.41	0.1987
ILMN_1241618	ILMN_218722	Flot1	1380.07	0.0000
ILMN_1257051	ILMN_223309	Glt25d2	119.14	0.0000
ILMN_2953411	ILMN_222370	Ppp3r1	2437.42	0.0000
ILMN_1248949	ILMN_192583	Gng12	93.92	0.0011
ILMN_2985228	ILMN_201543	Raet1a	20.59	0.1111
ILMN_3031402	ILMN_196771	Klra4	8.84	0.1976
ILMN_2721406	ILMN_220389	Crhr2	34.41	0.0288
ILMN_2640346	ILMN_213745	P2ry13	36.97	0.0342
ILMN_1231649	ILMN_184338	Vipr1	-5.22	0.6079
ILMN_2742197	ILMN_221929	Gabrb1	21.22	0.0908

ILMN_1230656	ILMN_186593	Grm2	13.42	0.1667
ILMN_2742576	ILMN_215618	Chrna4	8.26	0.2137
ILMN_2978444	ILMN_221904	Olfr1030	19.21	0.0940
ILMN_2997653	ILMN_255897	Olfr670	11.31	0.1848
ILMN_2629227	ILMN_212689	Olfr109	5.51	0.2553
ILMN_2438565	ILMN_186796	Pex1	-12.76	0.9519
ILMN_2607964	ILMN_210666	Aqp7	7.22	0.2126
ILMN_1242466	ILMN_218167	Psmb9	101.55	0.0000
ILMN_2683792	ILMN_195757	Ak5	27.87	0.0481
ILMN_2638349	ILMN_184209	Papss2	144.65	0.0000
ILMN_2803138	ILMN_261318	Prkaa2	200.45	0.0000
ILMN_2657409	ILMN_215266	Rps18	162.79	0.0000
ILMN_2899607	ILMN_214603	Ddx3y	12.92	0.1378
ILMN_1223689	ILMN_213898	Sf3a2	33.08	0.0438
ILMN_2956381	ILMN_222821	Syf2	2075.73	0.0000
ILMN_2898958	ILMN_214850	Hsd3b2	3959.33	0.0000
ILMN_1224967	ILMN_195714	Ltbp1	17.92	0.0833
ILMN_2767739	ILMN_210368	Slc2a2	-4.54	0.5000
ILMN_2669234	ILMN_216271	Dbh	25.83	0.0780
ILMN_1254488	ILMN_209990	Mgrn1	5561.33	0.0000
ILMN_2650013	ILMN_195614	Apc	-6.47	0.6132
ILMN_1237817	ILMN_221971	Gpr34	13.16	0.1699
ILMN_2694569	ILMN_218342	Cyp4b1	74.04	0.0011
ILMN_1255466	ILMN_205473	A830035A12Rik	20.67	0.1100
ILMN_2821371	ILMN_211776	Pet2	7.64	0.1923
ILMN_2736042	ILMN_221470	Cdh6	311.88	0.0000
ILMN_2755204	ILMN_213144	Slc28a3	39.05	0.0224
ILMN_1252016	ILMN_211921	Abhd2	40.05	0.0107
ILMN_2679974	ILMN_217182	D930042L22	17.04	0.1079
ILMN_2911454	ILMN_213499	5830457O10Rik	910.94	0.0000
ILMN_2634503	ILMN_213182	1700015E13Rik	30.51	0.0459
ILMN_1219038	ILMN_218215	Mill1	28.60	0.0598
ILMN_1219908	ILMN_184982	Tmem8	131.14	0.0000
ILMN_2795078	ILMN_189366	Unc93b1	150.15	0.0000
ILMN_1239632	ILMN_219631	Ng23	199.35	0.0000
ILMN_2743421	ILMN_222009	Slc29a2	95.77	0.0000
ILMN_2899578	ILMN_215838	Spert	20.59	0.0887
ILMN_2800813	ILMN_261748	Cabc1	28.27	0.0321
ILMN_1235237	ILMN_203257	8030462N17Rik	15.92	0.1175
ILMN_1225522	ILMN_186317	Trpc4ap	533.05	0.0000
ILMN_1256923	ILMN_207033	Mcf2l	6.97	0.2212
ILMN_3038743	ILMN_261549	Slc7a2	2.18	0.3259
ILMN_2744660	ILMN_209827	Igh-6	53.28	0.0075
ILMN_2612403	ILMN_211089	Mak10	1583.95	0.0000
ILMN_2780088	ILMN_221590	Tcp11l1	13.34	0.1528
ILMN_2742279	ILMN_221933	Defb19	2299.89	0.0000
ILMN_3162445	ILMN_230251	Trcg1	12.21	0.1453
ILMN_1242762	ILMN_207339	Lgtn	13.71	0.1207
ILMN_1226573	ILMN_216987	1700055M20Rik	-8.18	0.7361
ILMN_2744846	ILMN_222116	Guca2b	584.01	0.0000
ILMN_2596666	ILMN_209529	BC048403	52.05	0.0021
ILMN_2610981	ILMN_210954	Ostb	522.15	0.0000
ILMN_2686825	ILMN_217727	5430427O19Rik	13.69	0.1442

ILMN_1243741	ILMN_221378	Rora	217.36	0.0000
ILMN_1259830	ILMN_219587	1700092K14Rik	15.67	0.1314
ILMN_2977193	ILMN_220407	Adat1	155.02	0.0000
ILMN_1236379	ILMN_212078	Lcn3	14.99	0.1282
ILMN_2681419	ILMN_217283	2310061I04Rik	8.36	0.1987
ILMN_2416218	ILMN_184143	5530400B01Rik	108.36	0.0000
ILMN_1232604	ILMN_184761	Olfml2a	15.84	0.1314
ILMN_3153893	ILMN_254970	Zfp91	521.89	0.0000
ILMN_2731769	ILMN_221164	Plekhb2	941.55	0.0000
ILMN_2597987	ILMN_198171	Gpr116	68.43	0.0021
ILMN_2818294	ILMN_214124	Srpx2	96.30	0.0000
ILMN_2728567	ILMN_220917	5830403F22Rik	14.68	0.1303
ILMN_1232428	ILMN_218138	Rpp30	2537.88	0.0000
ILMN_1250303	ILMN_184717	C730029A08Rik	122.49	0.0000
ILMN_1254912	ILMN_188905	4930520P13Rik	3.55	0.2906
ILMN_1248532	ILMN_206830	Pcsk5	16.11	0.1250
ILMN_1239565	ILMN_213927	Ptprt	12.77	0.1741
ILMN_2729834	ILMN_221007	Clybl	23.90	0.0748
ILMN_2667396	ILMN_216104	Foxf1a	20.45	0.0876
ILMN_2438678	ILMN_186808	Treml4	10.29	0.1528
ILMN_1216036	ILMN_191849	Cnnm3	71.71	0.0011
ILMN_1215854	ILMN_212683	Matn4	5.28	0.2372
ILMN_1217118	ILMN_221027	Enpp5	1229.90	0.0000
ILMN_2608502	ILMN_210721	9530019H20Rik	71.05	0.0000
ILMN_2598519	ILMN_209718	Adamts20	9.12	0.1806
ILMN_2705276	ILMN_217879	Api5	0.55	0.3429
ILMN_2803921	ILMN_223061	Ly6d	46.23	0.0160
ILMN_1254452	ILMN_213468	A430093F15Rik	25.00	0.0652
ILMN_3162202	ILMN_259681	Tmem86b	11.48	0.1250
ILMN_2878742	ILMN_235218	Gm906	11.93	0.1677
ILMN_1216226	ILMN_223241	Cbln2	12.46	0.1549
ILMN_2723803	ILMN_220564	Serpinb12	17.12	0.1154
ILMN_2546984	ILMN_202098	2810006K23Rik	41.78	0.0278
ILMN_2693094	ILMN_209585	Lrch4	17.80	0.1090
ILMN_2591264	ILMN_208965	Orm2	11.19	0.1624
ILMN_3145636	ILMN_254386	Chd5	13.96	0.1357
ILMN_2580261	ILMN_206463	Edil3	12.86	0.1410
ILMN_1252742	ILMN_195491	Trpv5	245.10	0.0000
ILMN_2733290	ILMN_221272	Ivl	11.89	0.1603
ILMN_3162587	ILMN_209499	Kcna6	13.13	0.1549
ILMN_1227591	ILMN_209116	Sorcs1	6.83	0.2137
ILMN_2683757	ILMN_217485	Spnb4	-2.93	0.4530
ILMN_2859932	ILMN_243346	Fndc8	8.65	0.2019
ILMN_1259344	ILMN_217506	Prap1	56.21	0.0021
ILMN_1228723	ILMN_219473	Pcdhgb7	7.77	0.2019
ILMN_1243407	ILMN_208839	Klk10	11.87	0.1624
ILMN_2891104	ILMN_217409	Tmco4	157.48	0.0000
ILMN_1213049	ILMN_203403	Odz2	-2.53	0.5011
ILMN_1214767	ILMN_203467	Tulp3	25.04	0.0321
ILMN_1260325	ILMN_208755	Pabpc4	7338.12	0.0000
ILMN_2601090	ILMN_209986	Gps1	4235.01	0.0000
ILMN_1234533	ILMN_212555	Slc22a9	99.90	0.0000
ILMN_2625168	ILMN_212325	Gja8	7.69	0.2126

ILMN_2756846	ILMN_194792	Mro	53.02	0.0043
ILMN_2760531	ILMN_223190	1110001D15Rik	18.25	0.1047
ILMN_2603253	ILMN_210207	Casc1	21.67	0.0855
ILMN_2697334	ILMN_218563	5730522E02Rik	2.55	0.3066
ILMN_3117876	ILMN_226085	Chi3l3	21.01	0.0929
ILMN_3139380	ILMN_226707	Mlxip	-4.34	0.5118
ILMN_2947369	ILMN_210290	Ppp2r3c	685.82	0.0000
ILMN_2682763	ILMN_217398	4930451I11Rik	6.13	0.2350
ILMN_1257088	ILMN_211526	Prss12	127.90	0.0000
ILMN_1230695	ILMN_187583	Vps13d	14.59	0.1335
ILMN_2756550	ILMN_196243	Slc12a3	1369.89	0.0000
ILMN_3106706	ILMN_220561	Spag4l	12.27	0.1795
ILMN_2987965	ILMN_253156	Nxf3	2.26	0.3056
ILMN_2992965	ILMN_256249	Dkc1	-1.77	0.4423
ILMN_2752300	ILMN_222614	4930452B06Rik	421.88	0.0000
ILMN_2907788	ILMN_254330	Upk1a	285.96	0.0000
ILMN_2707870	ILMN_213462	Mx1	8.94	0.1998
ILMN_2751771	ILMN_221415	Slc17a3	13.40	0.1677
ILMN_3128529	ILMN_232453	Mn1	10.39	0.1944
ILMN_2829203	ILMN_257529	Fkbp6	2.05	0.3536
ILMN_2589525	ILMN_187076	Cpeb3	20.78	0.0876
ILMN_1255339	ILMN_187541	V1re7	10.24	0.1806
ILMN_1241916	ILMN_214943	4833423E24Rik	10.80	0.1635
ILMN_2746521	ILMN_222223	Icam5	20.00	0.0641
ILMN_3000416	ILMN_215375	Foxi1	52.14	0.0096
ILMN_2627523	ILMN_212533	BC024139	8.77	0.1838
ILMN_2735583	ILMN_221435	Agr2	10.94	0.1752
ILMN_2731523	ILMN_221143	Bat5	2743.17	0.0000
ILMN_2639296	ILMN_213649	Ndel1	2736.05	0.0000
ILMN_1222016	ILMN_219834	Slc5a10	1966.72	0.0000
ILMN_1217798	ILMN_206719	Adam22	1.06	0.3408
ILMN_2445049	ILMN_187546	Ttc15	12.67	0.1410

Table 3. Number of differentially detected genes (BH-adjusted p-value and 2-fold change) analyzing only genes with detection p-value<0.01.

	**Down regulated**	**Unchanged**	**Up regulated**
E14.5 Mutant vs. Control	1	11194	1
E17.5 Mutant vs. Control	14	11172	10
E17.5 Mutant vs.E14.5 Mutant	253	10353	590
E17.5 Control vs.E14.5 Control	217	10356	623

REFERENCES

1. Pandey P, Qin S, Ho J, Zhou J, Kreidberg JA (2011) Systems biology approach to identify transcriptome reprogramming and candidate microRNA targets during the progression of polycystic kidney disease. BMC Syst Biol 5: 56.
